# The associations between COVID-19 diagnosis, type 1 diabetes, and the risk of diabetic ketoacidosis: A nationwide cohort from the US using the Cerner Real-World Data

**DOI:** 10.1371/journal.pone.0266809

**Published:** 2022-04-19

**Authors:** Fares Qeadan, Benjamin Tingey, Jamie Egbert, Marcus G. Pezzolesi, Mark R. Burge, Kathryn A. Peterson, Trenton Honda

**Affiliations:** 1 Parkinson School of Health Sciences and Public Health, Loyola Univesity Chicago, Maywood, Illinois, United States of America; 2 Department of Internal Medicine, University of Utah School of Medicine, Salt Lake City, Utah, United States of America; 3 Department of Internal Medicine, University of New Mexico Hospital, Albuquerque, New Mexico, United States of America; 4 Department of Gastroenterology, University of Utah School of Medicine, Salt Lake City, Utah, United States of America; 5 School of Clinical and Rehabilitation Sciences, Bouvé College of Health Sciences, Northeastern University, Boston, Massachusetts, United States of America; Sohag University Faculty of Medicine, EGYPT

## Abstract

**Objective:**

To assess the risk of new-onset type 1 diabetes mellitus (T1D) diagnosis following COVID-19 diagnosis and the impact of COVID-19 diagnosis on the risk of diabetic ketoacidosis (DKA) in patients with prior T1D diagnosis.

**Research design and methods:**

Retrospective data consisting of 27,292,879 patients from the *Cerner Real-World Data* were used. Odds ratios, overall and stratified by demographic predictors, were calculated to assess associations between COVID-19 and T1D. Odds ratios from multivariable logistic regression models, adjusted for demographic and clinical predictors, were calculated to assess adjusted associations between COVID-19 and DKA. Multiple imputation with multivariate imputation by chained equations (MICE) was used to account for missing data.

**Results:**

The odds of developing new-onset T1D significantly increased in patients with COVID-19 diagnosis (OR: 1.42, 95% CI: 1.38, 1.46) compared to those without COVID-19. Risk varied by demographic groups, with the largest risk among pediatric patients ages 0–1 years (OR: 6.84, 95% CI: 2.75, 17.02) American Indian/Alaskan Natives (OR: 2.30, 95% CI: 1.86, 2.82), Asian or Pacific Islanders (OR: 2.01, 95% CI: 1.61, 2.53), older adult patients ages 51–65 years (OR: 1.77, 95% CI: 1.66, 1.88), those living in the Northeast (OR: 1.71, 95% CI: 1.61, 1.81), those living in the West (OR: 1.65, 95% CI: 1.56, 1.74), and Black patients (OR: 1.59, 95% CI: 1.47, 1.71). Among patients with diagnosed T1D at baseline (n = 55,359), 26.7% (n = 14,759) were diagnosed with COVID-19 over the study period. The odds of developing DKA for those with COVID-19 were significantly higher (OR 2.26, 95% CI: 2.04, 2.50) than those without COVID-19, and the largest risk was among patients with higher Elixhauser Comorbidity Index.

**Conclusions:**

COVID-19 diagnosis is associated with significantly increased risk of new-onset T1D, and American Indian/Alaskan Native, Asian/Pacific Islander, and Black populations are disproportionately at risk. In patients with pre-existing T1D, the risk of developing DKA is significantly increased following COVID-19 diagnosis.

## Introduction

Since the emergence of SARS COV-2 across the globe, a significant amount of evidence has shown those with diabetes mellitus (DM) to be at particularly high risk of morbidity and mortality from the virus. Just months after the World Health Organization declared a pandemic, case reports began emerging demonstrating the disproportionate impact of COVID-19 on people with diabetes, with one early report from Seattle demonstrating that nearly 60% of critically ill patients they observed had diabetes [1]. These findings were consistent with a recent meta-analysis of 33 studies from across the globe, which found pooled effect estimates for those with diabetes demonstrating a 90% increased risk of mortality and a 175% increased risk of a severe disease course [2]. DM, it seems, is a strong and significant risk factor for morbidity and mortality from COVID-19. Importantly, these prior studies either explore T2D only, or do not differentiate types of DM in their inclusion criteria. As such, it is not possible to determine whether and to what extent these observed associations are applicable to all DM types, or whether the associations and risks differ importantly by diabetes subtype.

Type 1 diabetes mellitus (T1D), which makes up approximately 5% of all DM, is an autoimmune disease that is differentiated from T2D by a unique pathophysiology, risk factors, and comorbidity profile [3]. While only a small number of studies, mostly case reports [4–8], have investigated associations between SARS-CoV-2 infection and T1D incidence, T1D has known etiologic and severity associations with a number of prior viral infections. For example, enterovirus infection has been found in a number of studies to confer increased risk of beta cell autoimmunity [9], and progression from beta cell autoimmunity to clinical T1D [10]. While the best evidence is available for enteroviruses, potential links have also been found for mumps [11], ebstein-barr virus [12], and cytomegalovirus [13]. Viral infection is thought to contribute to T1D incidence by the following pathophysiologic mechanisms: Viral molecular mimicry, direct effects of viral infection and replication in beta cells, and/or host inflammatory factors in response to infection, leading to beta cell apoptosis or cytotoxic-mediated cell death [14]. In addition to T1D incidence, prior literature has suggested that metabolic complications usually associated with T1D, such as diabetic ketoacidosis (DKA), may also be increased in SARS-CoV-2 infection [15–18]. However, these findings are not consistent throughout the literature. DKA is an important and deadly complication of T1D characterized by hyperglycemia, acidosis, elevated urine and serum ketone levels, and an elevated anion gap [19]. A study out of New York showed that while during the pandemic the volume of emergency department (ED) visits dropped by 29% from March 1 to May 31, 2020, the number of ED admissions for DKA increased by 70% [20]. This, taken together with a number of case reports [15, 17, 21, 22] from across the globe demonstrating increased DKA risk with SARS-CoV-2 infection, suggest that COVID-19 may impact T1D morbidity and mortality through increased risk of this important metabolic complication.

We aim to examine the associations between COVID-19 diagnosis and incident T1D in a national US cohort, as well as whether and to what extent COVID-19 diagnosis increases the risk of DKA among those with established T1D.

## Research design and methods

### Settings and participants

**Fig 1:**
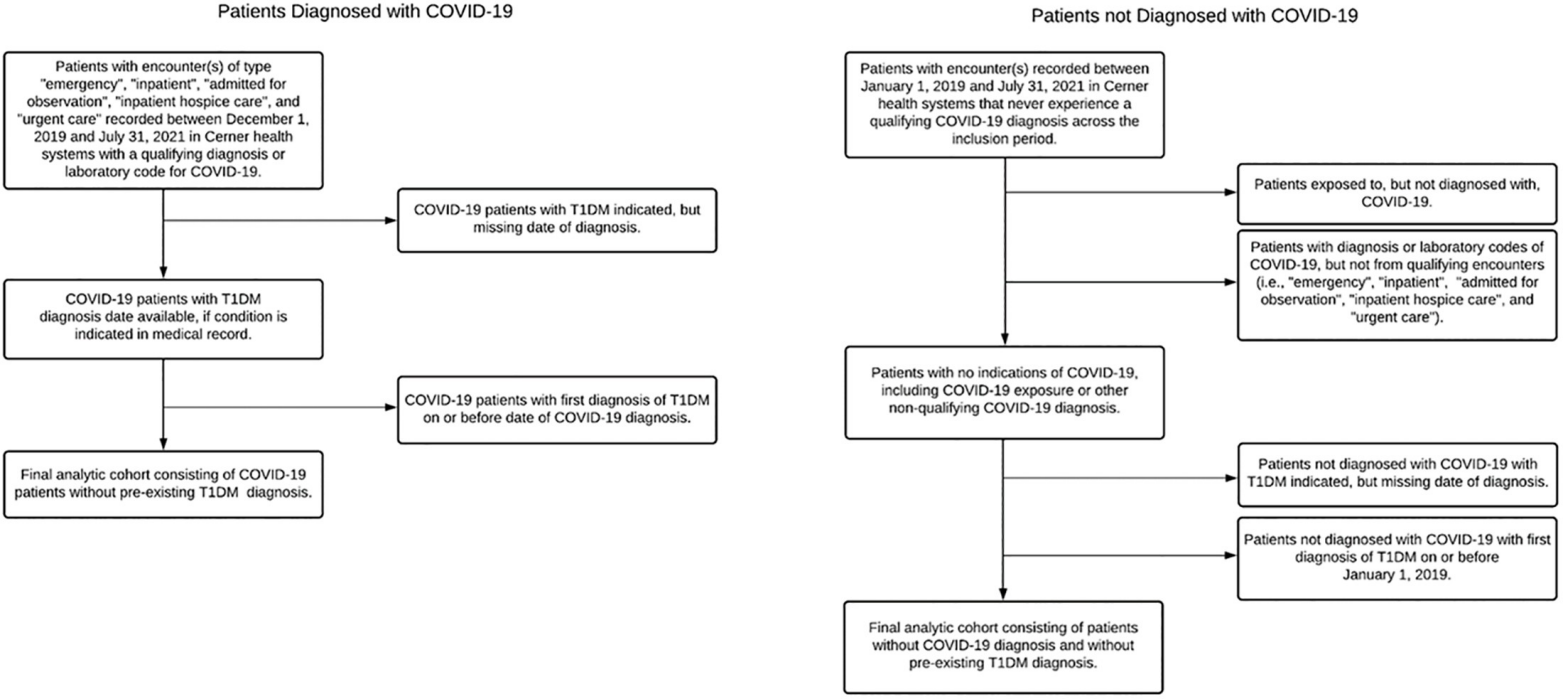
Inclusion and exclusion criteria used to generate the final analytic cohort consisting of patients without pre-existing T1D diagnosis. Data from were used to examine the associations between COVID-19 diagnosis and incident T1D in the US.

**Fig 2:**
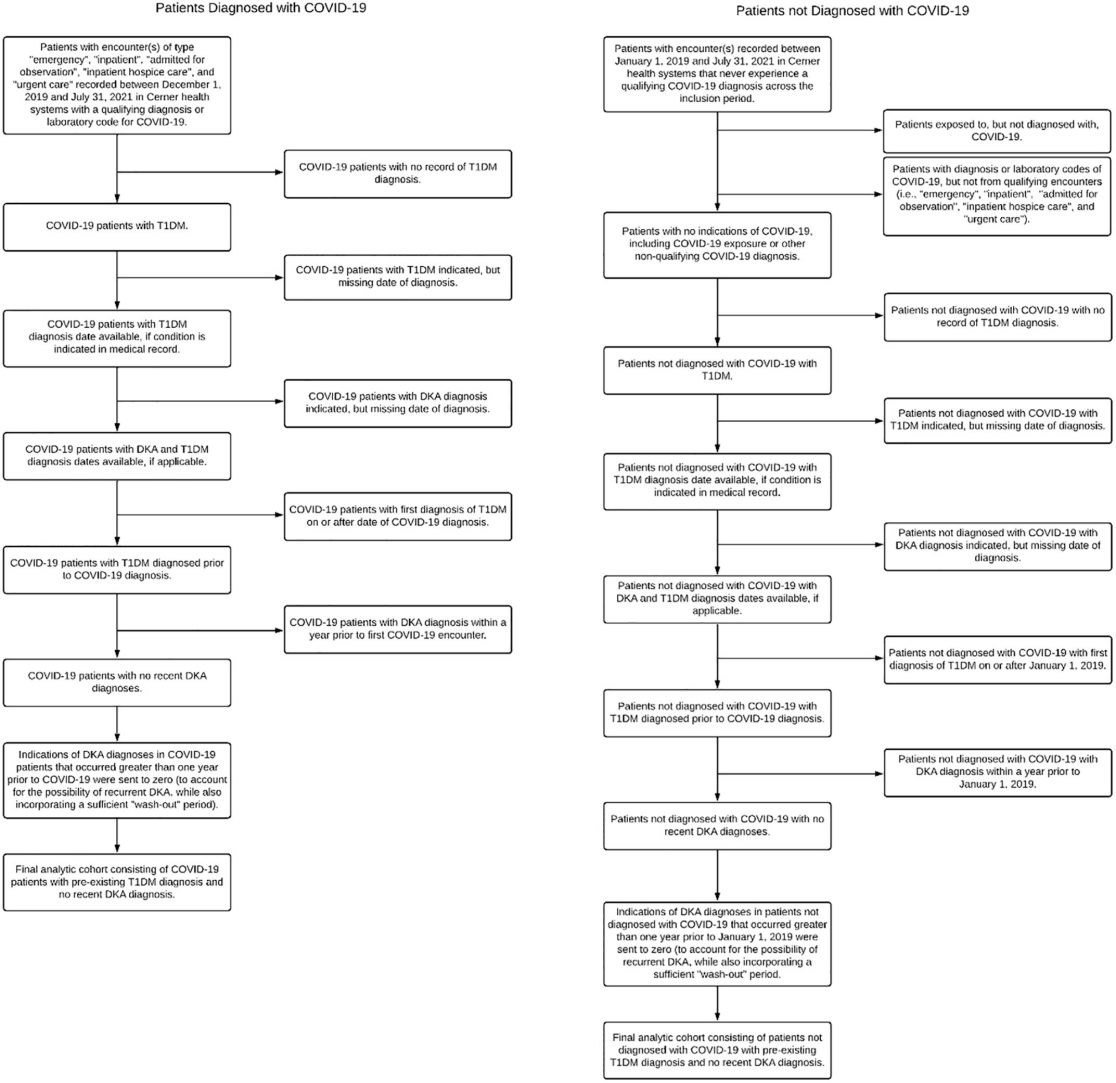
Inclusion and exclusion criteria used to generate the final analytic cohort consisting of patients with pre-existing T1D, no recent DKA diagnosis, non-recent DKA indications removed, and appropriate diagnosis dates known. Data were used to assess the extent to which COVID-19 diagnosis increases the risk of DKA among those with pre-existing T1D.

The retrospective study cohorts were obtained from Cerner Real-World Data [22]. Cerner contains longitudinal electronic health records (EHRs) of nearly 100 million unique patients from 113 contributing U.S. health systems, with roughly 1.4 billion encounters as of October 2021. “*Data in Cerner Real-World Data ™ is extracted from the electronic health records (EHR) of hospitals and clinics who have consented to such use*. *Encounters may include pharmacy*, *clinical and microbiology laboratory*, *admission and billing information from affiliated patient care locations*. *All admissions*, *medication orders and dispensing*, *laboratory orders and specimens are date and time stamped*, *providing a temporal relationship between treatment patterns and clinical information*. *Cerner de-identifies Cerner Real-World Data in compliance with Health Insurance Portability and Accountability Act*” [23]. Patients eligible for analysis included those who had a confirmed diagnosis code of COVID-19 or a positive SARS-CoV-2 lab result at encounters from December 1, 2019 through July 31, 2021, of type: “emergency”, “inpatient”, “admitted for observation”, “inpatient hospice care”, or “urgent care.” Patients with qualifying diagnosis codes or labs but not any from the above qualifying encounter types were removed. Patients only exposed to COVID-19, but lacking confirmed diagnoses (as determined by diagnostic codes and lab results) were also removed from the analysis. Additionally, all other patients treated in the same health systems as these COVID-19 patients, seen at least once since January 1, 2019 and until July 31, 2021, were included in the study. Patients not seen since before 2019 were removed. This extra window of inclusion for non-COVID patients was provided to capture normal hospital trends before the pandemic occurred. After inclusion, patients were immediately studied over time for development of outcomes. An additional two months of follow-up was provided for both COVID and non-COVID patients, extending until September 30, 2021, to assess outcomes for those included near the end of the study period. Of these eligible patients, two cohorts were studied that involved: 1) patients with no history of T1D at the beginning of the study period and 2) patients with a history of T1D at the beginning of the study period. Inclusion and exclusion criteria used to generate the two cohorts described above are found in Figs 1 and 2, respectively. The University of Utah Institutional Review Board (IRB #136696) determined this study to be exempt and thus waived the requirements for ethical approval and informed consent.

### Outcomes

For those with no history of T1D at baseline, the primary outcome of interest was a new diagnosis of T1D defined as new presence of T1D associated ICD-10 codes within the medical record at least 24 hours after the date of confirmed COVID-19 diagnosis, or at least 24 hours after January 1, 2019 for those without COVID-19 diagnosis (S1 Table in S1 File). For those with a history of T1D at baseline, the primary outcome of interest was a new diagnosis of DKA defined as new incidence of DKA associated ICD-10 codes within the medical record at least 24 hours after the date of confirmed COVID-19 diagnosis, or at least 24 hours after January 1, 2019 for those without COVID-19 diagnosis (S1 Table in S1 File). These were binary indications (yes/no).

### Predictors

The primary exposure of interest for both cohorts was a binary indication of COVID-19 diagnosis. Other baseline demographic predictor variables for both cohorts included the continuous age (in years) and the categorical age groups (0–1, 2–5, 6–12, 13–17, 18–35, 36–50, 51–65, >65), gender (female, male, other), race and ethnicity (non-Hispanic [NH] American Indian or Alaskan Native [AI/AN], NH Asian or Pacific Islander [API], NH Black, Hispanic/Latino, and NH White), marital status (married/partner, not married) as a proxy for social support, and US geographical region (Northeast, Southeast, Midwest, West). Clinical predictor variables were only used for those with a history of T1D at baseline, and included baseline patient comorbidity (<0, 0, 1–4, >= 5) [24]. Comorbidity was measured by the Elixhauser comorbidity index (ECI) and was weighted using the Agency for Healthcare Research and Quality (AHRQ) methodology [24]. Higher scores indicate higher disease burden and lower scores indicate lower disease burden. A continuous version of the ECI was also provided. The specific conditions used in the ECI calculation, with accompanying ICD-10 codes, are displayed in S2 Table in S1 File. Conditions were searched upon extending from baseline back until October 2015, which was the implementation month/year of ICD-10. Other clinical predictors included binary indications of diabetes technology use, such as continuous glucose monitoring (CGM) and insulin pumps. If such use occurred more than 6 months prior to COVID-19 diagnosis (or 6 months prior to January 1, 2019 for controls), then those indications were classified as “no.” String matching as well as codes were used to identify these clinical predictors, and the codes are listed in S3 Table in S1 File. Final clinical predictors included the duration of T1D (time in years from T1D diagnosis to either COVID-19 diagnosis date or January 1, 2019) and baseline hemoglobin A1c (HbA1c) test percentage (taken as the most recent monthly averaged result within 6 months prior to December 1, 2019 for COVID patients or 6 months prior to January 1, 2019 for non-COVID patients). The final date of inclusion of HbA1c for COVID patients was November 30, 2019 so as to be sure that the results were not conflated with an induction period of undiagnosed COVID-19. String matching was used to identify all HbA1c labs.

### Additional measures

An additional outcome variable was the incidence rate of DKA per 100,000 patients. This varied by each month, and provided the count of T1D patients newly diagnosed with DKA divided by the count of T1D patients not yet experiencing a DKA diagnosis, per 100,000 patients. Each ensuing month, patients that had experienced a diagnosis of DKA in the previous month were removed from the denominator. The additional exposure was time in months.

### Statistical analysis

The analysis sought to answer two research questions: 1) What is the association between COVID-19 diagnosis and development of T1D in patients without a history of T1D? 2) What is the association between COVID-19 diagnosis and development of DKA in patients with a history of T1D? To answer the first question, overall demographics were presented for patients without a history of T1D at baseline. Variables, being categorical, were presented with frequencies and percentages. Characteristics were stratified by those with and without COVID-19 diagnosis and compared with Chi-Squared tests. Overall T1D incidence was also compared between those with and without COVID-19 diagnosis. Frequencies and percentages were presented, along with an odds ratio (OR) and 95% confidence interval (CI). The OR calculated the odds of T1D diagnosis for those diagnosed with COVID-19 compared to those without COVID-19 diagnosis. Significance of association was determined by a Chi-Squared test. This comparison was stratified by demographic groups (age, gender, race and ethnicity, marital status, and US geographical region). For small sample sizes, Fisher’s Exact test was used to determine the significance of association.

To answer the second question, overall demographic and clinical characteristics were presented for patients with a history of T1D at baseline. Normally distributed continuous variables were presented with means and standard deviations, and non-normally distributed continuous variables were presented medians and interquartile ranges (Q1, Q3). Categorical variables were presented with frequencies and percentages. Characteristics were again stratified by those with and without COVID-19 diagnosis and compared with two-sample t-tests for normally distributed continuous variables, Wilcoxon Rank-Sum tests for non-normally distributed continuous variables, and Chi-Squared tests for categorical variables. The incidence rate of DKA per 100,000, across each month of the study period, was presented in a line plot. Different lines were drawn for those with and without COVID-19. Variability was captured with 95% exact Poisson confidence intervals.

To address concerns related to missing data on HbA1c, even with a wide time frame of capture, Little’s missing completely at random (MCAR) test [25] was conducted to determine if the missing pattern was MCAR or not. Upon test results rejecting the assumption of MCAR, multiple imputation with multivariate imputation by chained equations (MICE) [26] was conducted. Five separate imputed datasets were provided, each with a predictive mean matching (PMM) method to impute the continuous HbA1c value using all other variables included in later modeling. Imputation diagnostics were assessed, including distributional similarities between original and imputed data. Adjusted associations of COVID-19 with development of DKA were calculated in a logistic regression model, that pooled all five imputed dataset models together using Rubin’s rules [27]. The model was adjusted for age, gender, race and ethnicity, marital status, US geographical region, ECI, insulin pump use, CGM use, diabetes duration, and baseline HbA1c. Adjusted ORs (aORs) were provided with 95% CIs constructed from a profile likelihood and p-values constructed from a z-statistic. Model diagnostics and variable multicollinearity was assessed. As a sensitivity analysis, models were repeated (without imputation) by modeling only for the complete cases (only rows of data where HbA1c was not missing) as well as fitting the model without HbA1c. Model coefficients were compared with that of the multiple imputed results. Whether or not HbA1c was missing at random (MAR) or missing not at random (MNAR), these sensitivity analyses were conducted to ensure bias was not introduced.

Finally, to assess the impact of disease burden and COVID-19 diagnosis on the development of DKA, model predicted probabilities of DKA were plotted against the ECI. Different lines were again drawn for those with and without COVID-19 diagnosis by using a locally estimated scatterplot smoothing (LOESS) curve technique [28]. Probabilities were captured from the final pooled model, which in many cases is equivalent to capturing probabilities from each imputed dataset and then pooling them manually [29].

P-values less than 0.05 were considered statistically significant and all statistical tests were two-sided. Analyses were conducted using R version 4.0.2 (R Foundation for Statistical Computing).

### Additional note on missing data

Because Cerner provides longitudinal data, multiple rows of data were available for patients across multiple tables. If patients were missing certain information in one table, that information was searched upon across multiple other tables and filled in, when possible, for the final analysis cohort. When data could not be obtained, imputation methods (as described previously) were performed.

## Results

Table 1 shows the demographic characteristics of patients without history of T1D at the beginning of the study period. The study population included 27,292,879 individuals at baseline, of whom 9.1% (n = 2,489,266) were diagnosed with COVID-19 within the study period. The majority (54.1%) were female, white (48.3%), and not married (68.1%). Compared to patients not diagnosed with COVID-19, COVID-19 patients were significantly older, and had significantly higher percentages of males, Hispanic/Latinos, and patients from the Southeast region (all P<0.001).

**Table 1:** Demographic characteristics of patients with no history of T1D (overall and stratified by COVID-19 diagnosis).

	Total	COVID-19	Non COVID-19	p-value^‡^
		n (%)*	n (%)*	
**Total**	27,292,879	2,489,266 (9.1^†^)	24,803,613 (90.9^†^)	
**Age (Years)**				**<0.001**
0–1	668,649 (2.6)	64,225 (2.7)	604,424 (2.6)	
2–5	1,505,453 (5.9)	106,019 (4.5)	1,399,434 (6.0)	
6–12	2,040,946 (8.0)	108,300 (4.6)	1,932,646 (8.3)	
13–17	1,609,468 (6.3)	96,555 (4.1)	1,512,913 (6.5)	
18–35	5,837,610 (22.9)	552,873 (23.6)	5,284,737 (22.8)	
36–50	4,320,970 (16.9)	405,313 (17.3)	3,915,657 (16.9)	
51–65	4,608,216 (18.1)	438,083 (18.7)	4,170,133 (18.0)	
>65	4,905,768 (19.2)	575,750 (24.5)	4,330,018 (18.7)	
**Gender**				**<0.001**
Female	13,755,616 (54.1)	1,264,069 (53.9)	12,491,493 (54.1)	
Male	11,661,129 (45.9)	1,081,608 (46.1)	10,579,475 (45.9)	
**Race and Ethnicity**				**<0.001**
NH^§^-AI/AN^||^	229,503 (0.8)	33,588 (1.3)	195,914 (0.8)	
NH-API^#^	688,752 (2.5)	51,872 (2.1)	636,878 (2.6)	
NH-Black	2,494,252 (9.1)	305,481 (12.3)	2,188,759 (8.8)	
Hispanic/Latino	3,790,390 (13.9)	534,419 (21.5)	3,255,950 (13.1)	
NH-White	13,192,433 (48.3)	1,197,043 (48.1)	11,995,342 (48.4)	
NH Other/Unknown	6,897,548 (25.3)	366,863 (14.7)	6,530,770 (26.3)	
**Marital Status**				**<0.001**
Married/Partner	7,263,907 (31.9)	725,508 (31.4)	6,538,399 (31.9)	
Not Married	15,534,382 (68.1)	1,581,719 (68.6)	13,952,663 (68.1)	
**Region**				**<0.001**
Northeast	5,479,188 (21.4)	464,865 (19.8)	5,014,323 (21.6)	
Southeast	5,240,882 (20.5)	534,753 (22.8)	4,706,129 (20.3)	
Midwest	6,985,309 (27.3)	627,094 (26.7)	6,358,215 (27.4)	
West	7,839,965 (30.7)	720,894 (30.7)	7,119,071 (30.7)	

* counts (may not sum up to total due to removal of missing rows), column %’s;

^†^ % out of total (27,292,879);

^‡^ Chi-square test;

^§^ Non-Hispanic;

^||^ American Indian/Alaskan Native;

^#^ Asian/Pacific Islander.

Table 2 shows the associations between COVID-19 diagnosis and incidence of T1D during the study period overall and stratified by participant characteristics. Among all participants, COVID-19 diagnosis was associated with a 42% increased odds of developing new-onset T1D (OR: 1.42, 95% CI: 1.38, 1.46). Risks varied by age group, with the highest risk among pediatric patients ages 0–1 years (OR 6.84, 95% CI: 2.75, 17.02), 2–5 years (OR 2.19, 95% CI: 1.68, 2.85), 6–12 years (OR 2.04, 95% CI: 1.78, 2.33), and 13–17 years (OR 1.56, 95% CI: 1.38, 1.76). Those aged 18–35 years with COVID-19 saw no difference in risk of T1D compared to those not diagnosed with COVID-19 (OR 0.97, 95% CI: 0.97, 1.04). However, risk increased significantly again across older adult age groups for those 36–50 years (OR 1.54, 95% CI: 1.44, 1.64), 51–65 years (OR 1.77, 95% CI: 1.66, 1.88), and those >65 years (OR 1.43, 95% CI: 1.34, 1.52). Males had slightly higher risk (OR: 1.49, 95% CI: 1.42, 1.55) than females (OR: 1.36, 95% CI: 1.30, 1.42). Differences were observed by race/ethnicity, with the largest risks among AI/AN (OR: 2.30, 95% CI: 1.86, 2.82), followed by API (OR: 2.01, 95% CI: 1.61, 2.53), Black (OR: 1.59, 95% CI: 1.47, 1.71), Hispanic (OR: 1.52, 95% CI: 1.41, 1.63), and White (OR: 1.18, 95% CI: 1.13, 1.23) individuals. Risks also differed by geographic region, with the highest risk observed among those living in the Northeast (OR: 1.71, 95% CI: 1.61, 1.81), and no risk difference among those living in the Southeast (OR: 0.98, 95% CI: 0.91, 1.05). Risks were both similarly elevated among those married and not/married.

**Table 2:** Association of COVID-19 diagnosis with incidence of T1D among patients in Cerner.

		Type 1 D Diagnosis		
Overall	Total Patients	n (%*)	OR^†^ (95% CI)	p-value^‡^
Non COVID-19	24,803,613	36,348 (0.15)	REF = 1	**<0.001**
COVID-19	2,489,266	5,163 (0.21)	1.42 (1.38, 1.46)	
**Stratified**				
**0–1**				**<0.001** ^§^
Non COVID-19	604,424	11 (0.00)	REF = 1	
COVID-19	64,225	8 (0.01)	6.84 (2.75, 17.02)	
**2–5**				**<0.001**
Non COVID-19	1,399,434	392 (0.03)	REF = 1	
COVID-19	106,019	65 (0.06)	2.19 (1.68, 2.85)	
**6–12**				**<0.001**
Non COVID-19	1,932,646	2,113 (0.11)	REF = 1	
COVID-19	108,300	241 (0.22)	2.04 (1.78, 2.33)	
**13–17**				**<0.001**
Non COVID-19	1,512,913	2,860 (0.19)	REF = 1	
COVID-19	96,555	284 (0.29)	1.56 (1.38, 1.76)	
**18–35**				0.40
Non COVID-19	5,284,737	9,864 (0.19)	REF = 1	
COVID-19	552,873	1,003 (0.18)	0.97 (0.91, 1.04)	
**36–50**				**<0.001**
Non COVID-19	3,915,657	6,351 (0.16)	REF = 1	
COVID-19	405,313	1,010 (0.25)	1.54 (1.44, 1.64)	
**51–65**				**<0.001**
Non COVID-19	4,170,133	6,386 (0.15)	REF = 1	
COVID-19	438,083	1,188 (0.27)	1.77 (1.66, 1.88)	
**>65**				**<0.001**
Non COVID-19	4,330,018	5,829 (0.09)	REF = 1	
COVID-19	575,750	1,106 (0.20)	1.43 (1.34, 1.52)	
**Female**				**<0.001**
Non COVID-19	12,491,493	16,283 (0.13)	REF = 1	
COVID-19	1,264,069	2,245 (0.18)	1.36 (1.30, 1.42)	
**Male**				**<0.001**
Non COVID-19	10,579,475	17,488 (0.17)	REF = 1	
COVID-19	1,081,608	2,653 (0.25)	1.49 (1.42, 1.55)	
**NH-AIAN**				**<0.001**
Non COVID-19	195,914	318 (0.16)	REF = 1	
COVID-19	33,588	125 (0.37)	2.30 (1.86, 2.82)	
**NH-API**				**<0.001**
Non COVID-19	636,878	536 (0.08)	REF = 1	
COVID-19	51,872	88 (0.17)	2.01 (1.61, 2.53)	
**NH-Black**				**<0.001**
Non COVID-19	2,188,759	3,567 (0.16)	REF = 1	
COVID-19	305,481	789 (0.26)	1.59 (1.47, 1.71)	
**Hispanic**				**<0.001**
Non COVID-19	3,255,950	3,831 (0.12)	REF = 1	
COVID-19	534,419	954 (0.18)	1.52 (1.41, 1.63)	
**NH-White**				**<0.001**
Non COVID-19	11,995,342	21,693 (0.18)	REF = 1	
COVID-19	1,197,043	2,558 (0.21)	1.18 (1.13, 1.23)	
**Married/Partner**				**<0.001**
Non COVID-19	6,538,399	10,207 (0.16)	REF = 1	
COVID-19	725,508	1,523 (0.21)	1.34 (1.27, 1.42)	
**Not Married**				**<0.001**
Non COVID-19	13,952,663	21,844 (0.16)	REF = 1	
COVID-19	1,581,719	3,329 (0.21)	1.35 (1.30, 1.39)	
**Northeast**				**<0.001**
Non COVID-19	5,014,323	8,599 (0.17)	REF = 1	
COVID-19	464,865	1,360 (0.29)	1.71 (1.61, 1.81)	
**Southeast**				0.56
Non COVID-19	4,706,129	7,593 (0.16)	REF = 1	
COVID-19	534,753	844 (0.16)	0.98 (0.91, 1.05)	
**Midwest**				**<0.001**
Non COVID-19	6,358,215	8,338 (0.13)	REF = 1	
COVID-19	627,094	1,157 (0.19)	1.41 (1.32, 1.50)	
**West**				**<0.001**
Non COVID-19	7,119,071	9,280 (0.13)	REF = 1	
COVID-19	720,894	1,545 (0.21)	1.65 (1.56, 1.74)	

* row %’s;

^†^ odds ratio;

^‡^ Chi-Squared test (except where otherwise noted);

^§^ Fisher’s exact test.

**Fig 3:**
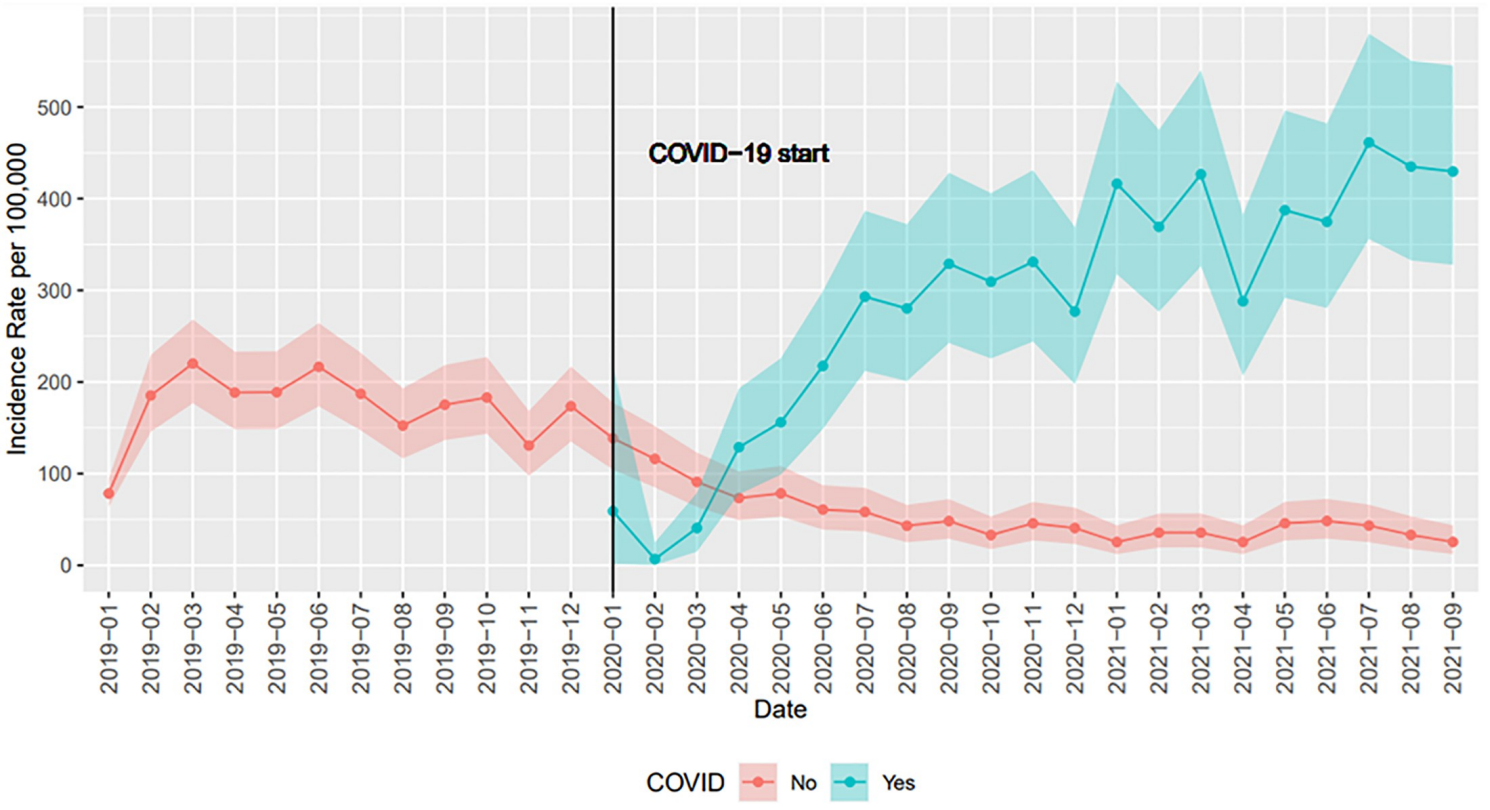
Incidence rate per 100,000 of DKA diagnosis from January 2019 to September 2021 (by COVID-19 status) among patients with previous T1D diagnosis.

Table 3 shows the demographic characteristics of those with T1D (n = 55,359) at baseline. Of these, 26.7% (n = 14,759) were diagnosed with COVID-19 over the study time period. Those diagnosed with COVID-19 were disproportionately Hispanic/Latino (19.5%), Black (15.9%), and with a ≥ 5 ECI (58.8%). Those diagnosed with COVID-19 had higher use of insulin pumps and CGM, longer time diagnosed with diabetes, and higher baseline HbA1c than those not diagnosed with COVID-19. Although the incidence rate of DKA per 100,000 people was elevated prior to the onset of COVID-19, those diagnosed with COVID-19 experienced a drastically higher incidence rate than ever achieved before the pandemic. COVID-19 patients continued to experience higher incidence than those without COVID-19 (Fig 3).

**Table 3:** Demographic and clinical characteristics of patients previously diagnosed with T1D (overall and stratified by COVID-19 diagnosis).

	Total	COVID-19	Non COVID-19	p-value^||^
		n (%*)	n (%*)	
**Total**	55,359 (100.0)	14,759 (26.7^†^)	40,600 (73.3^†^)	
**Age (Years)** ^‡^	45.4 (22.0)	49.9 (20.8)	43.8 (22.2)	**<0.001** ^#^ ^|^
**Gender**				**<0.001**
Female	28,032 (50.6)	7,652 (51.8)	20,380 (50.2)	
Male	27,327 (49.4)	7,107 (48.2)	20,220 (49.8)	
**Race and Ethnicity**				**<0.001**
NH-AI/AN	638 (1.2)	263 (1.8)	375 (0.9)	
NH-API	836 (1.5)	191 (1.3)	645 (1.6)	
NH-Black	6,743 (12.2)	2,349 (15.9)	4,394 (10.8)	
Hispanic/Latino	8,368 (15.1)	2,879 (19.5)	5,489 (13.5)	
NH-Other	3,899 (7.0)	1,000 (6.8)	2,899 (7.1)	
NH-White	34,875 (63.0)	8,077 (54.7)	26,798 (66.0)	
**Marital Status**				0.18
Married/Partner	17,750 (32.1)	4,798 (32.5)	12,952 (31.9)	
Not Married	37,609 (67.9)	9,961 (67.5)	27,648 (68.1)	
**Region**				**<0.001**
Northeast	13,100 (23.7)	3,923 (26.6)	9,177 (22.6)	
Southeast	9,645 (17.4)	2,450 (16.6)	7,195 (17.7)	
Midwest	15,641 (28.3)	3,937 (26.7)	11,704 (28.8)	
West	16,973 (30.7)	4,449 (30.1)	12,524 (30.8)	
**ECI Categorized**				**<0.001**
<0	8,051 (14.5)	2,020 (13.7)	6,031 (14.9)	
0	22,788 (41.2)	2,768 (18.8)	20,020 (49.3)	
1–4	3,898 (7.0)	1,290 (8.7)	2,608 (6.4)	
>=5	20,622 (37.3)	8,681 (58.8)	11,941 (29.4)	
**Insulin pump**	26,498 (47.9)	10,002 (67.8)	16,496 (40.6)	**<0.001**
**Continuous glucose monitoring**	4,024 (7.3)	1,314 (8.9)	2,710 (6.7)	**<0.001**
**Duration of diabetes (Years)** ^§^	2.1 (1.01, 3.0)	2.7 (1.4, 4.1)	1.9 (0.9, 2.8)	**<0.001** **
**Baseline HbA1c** ^‡^	8.6 (2.1)	9.1 (2.5)	8.5 (2.0)	**<0.001** ^#^ ^|^

* column %’s;

^†^ % out of total (55,359);

^‡^ mean (SD);

^§^ median (Q1, Q3);

^||^ Chi-square test (unless otherwise noted);

^#^ two-sample t-test (assuming equal variances);

** Wilcoxon Rank-Sum test.

**Fig 4:**
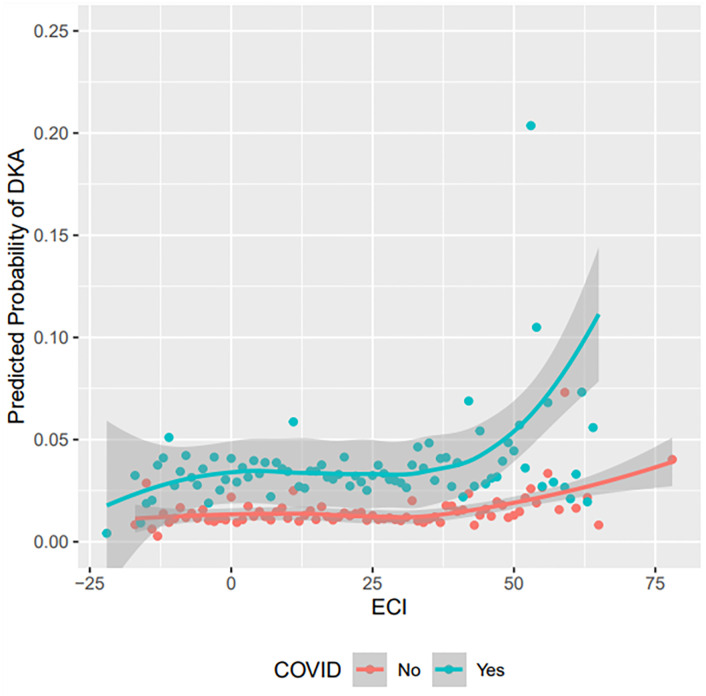
Predicted probability of DKA vs. ECI (by COVID-19 status) among patients with previous diagnosis of T1D.

Little’s MCAR test revealed a test statistic of 1522.95 and ensuing p-value of <0.001, which rejected the null hypothesis of HbA1c being MCAR. Table 4 shows the adjusted associations (pooled logistic regression coefficients from the multiple imputation with MICE) between COVID-19 diagnosis and incident DKA among participants with pre-existing T1D. Patients with COVID-19 had a 126% increased odds of developing DKA (aOR 2.26, 95% CI: 2.04, 2.50). The highest risks were seen among those living in the West (aOR 1.34, 95% CI: 1.19, 1.51). Hispanics and NH other races had lower odds of DKA than NH White patients, and those not married had higher odds of DKA (aOR 1.28, 95% CI: 1.13, 1.45) than those married. An elevated ECI was also associated with higher risks, such that in linear models a one unit increase in ECI was associated with a 2% increased odds of DKA (aOR 1.02, 95% CI: 1.01, 1.03), while in non-linear models (LOESS) the higher ECIs saw larger predicted probabilities of DKA (Fig 4). Those using insulin pumps had 57% lower odds of DKA (aOR 0.43, 95% CI: 0.39, 0.47) than those not using, and those using CGM had 25% lower odds of DKA (aOR 0.75, 95% CI: 0.62, 0.91) than those not using. For each one unit increase in baseline HbA1c percentage, the odds of DKA increased by 21% (aOR 1.21, 95% CI: 1.18, 1.24). When comparing the final adjusted associations to that of the complete case results and results unadjusted for HbA1c, all aORs were comparably similar.

**Table 4:** Adjusted associations of COVID-19 diagnosis with incidence of DKA among patients with previous T1D diagnosis.

	DKA Diagnosis		
	aOR*	95% CI	p-value
**COVID-19**			
No	REF = 1	REF = 1	-
Yes	2.26	(2.04, 2.50)	**<0.001**
**Age (5 year increments)**	0.84	(0.83, 0.85)	**<0.001**
**Gender**			
Female	REF = 1	REF = 1	-
Male	0.98	(0.89, 1.06)	0.57
**Race and Ethnicity**			
NH-White	REF = 1	REF = 1	-
NH-AI/AN	0.69	(0.48, 1.00)	0.052
NH-API	0.76	(0.51, 1.12)	0.17
NH-Black	0.89	(0.78, 1.02)	0.10
Hispanic/Latino	0.74	(0.65, 0.84)	**<0.001**
NH-Other	0.65	(0.53, 0.78)	**<0.001**
**Marital Status**			
Married/Partner	REF = 1	REF = 1	-
Not Married	1.28	(1.13, 1.45)	**<0.001**
**Region**			
Northeast	REF = 1	REF = 1	-
Southeast	0.66	(0.57, 0.76)	**<0.001**
Midwest	0.70	(0.61, 0.81)	**<0.001**
West	1.34	(1.19, 1.51)	**<0.001**
**ECI**	1.02	(1.01, 1.03)	**<0.001**
**Insulin pump**			
No	REF = 1	REF = 1	-
Yes	0.43	(0.39, 0.47)	**<0.001**
**Continuous glucose monitoring**			
No	REF = 1	REF = 1	-
Yes	0.75	(0.62, 0.91)	**0.004**
**Duration of diabetes (Years)**	1.00	(0.99, 1.01)	0.61
**Baseline HbA1c**	1.21	(1.18, 1.24)	**<0.001**

* adjusted odds ratio.

## Discussion

Ours is the first study of a national US cohort to examine associations between COVID-19 diagnosis and incident T1D among those without a history of T1D. We found strong and consistent associations suggesting that COVID-19 diagnosis indeed is associated with increased risk of new-onset T1D, and that American Indian/Alaskan Native, Asian/Pacific Islander, and Black populations are at a disproportionately high risk. Additionally, pediatric and older adult patients with COVID-19 have higher risks of T1D while those 18–35 had no differences. We are also the first to identify in a national US cohort of T1D patients an increased DKA risk among those with COVID-19, with those individuals with the highest comorbidity index at particularly high risk.

While we find strong associations between COVID-19 and incident T1D, to date few studies have investigated this potential link. In 2020, a UK study examining the Spring/Summer of 2020 reported a nearly 2-fold increase in the observed rate of T1D [30]. This is remarkable, as T1D diagnoses usually follow a seasonal pattern, with decreases in incidence in the Spring and Summer months in the Northern hemisphere [31]. However, there was inconsistent confirmation of COVID-19 status in this study to potentially link these additional cases with SARS CoV-2 infection. A study of pediatric T1D in Germany found a nominal increase in T1D rates among males (Observed: 28.1, 95% CI: 25.1, 33.1; Expected: 23.1, 95% CI: 20.8, 25.7) but a nominal decreased rates among females [32]. Similarly, an Italian survey of pediatric diabetes centers also reported a 23% decrease in new T1D diagnoses in 2020 relative to 2019 [16]. However, both of these studies are limited by their short observation durations since the pandemic’s onset (approximately 2 months in early 2020 for each) as well as delays in presentation due to lock-downs [33]. Other studies suggest that T1D or T1D-like diseases increased in prevalence during the pandemic. For example, a case series from Lincoln Hospital in New York, USA found T1D and T1D-like hyperglycemia to increase during the pandemic [4], as have case reports from New Jersey, USA [5], Peru [6], and France [34]. Of note, a number of these have demonstrated that COVID-associated DM is not always persistent across time, but occasionally dissipates weeks and months after the SARS-CoV-2 infection subsides [4, 34]. It is, however, unclear how common this disease trajectory is at this time.

Importantly, we found the associations between COVID-19 and T1D to differ by race, with the largest risks among American Indian/Alaskan Natives followed by Asian/Pacific Islanders, Black, Hispanic, and White patients. This may reflect differential risk trajectories of T1D by race/ethnicity that preexisted the pandemic. For example, one study found that between 2011 and 2015, the incidence of T1D increased among NH Black patients by 4% per year (95% CI: 1.7, 6.3), Hispanics by 2.5% per year 95% CI: 0.5, 4.6), and Asian/Pacific Islander patients by 8.5% per year (95% CI: 3.2, 14.0), while NH White patients did not see significant annual increases (0.5, 95% CI: -0.7, 1.7) [35]. The reasons for these differences by race/ethnicity remain unclear.

While consistent with our *a priori* expectations we observed the largest effect esmiates by far in pediatric patients, it was surprising to find increased risk of T1D among older adults as well. A number of potential explanations for this exist. First, while pediatric age is a strong risk factor for T1D, approximately 25% of cases will ultimately be diagnosed in adults, with known risk extending into the oldest old age groups [36, 37]. Second, at least one prior case series describing COVID-associated DM presenting with T1D-like presentations described this phenomenon only among adults (Median age 54 years), which is consistent with our findings [4]. Third, given the well documented differences in clinical presentation and severity of SARS-CoV-2 infection among pediatric patients, and the progressively increasing severity seen in advancing ages, it is possible that more severe pathologic effects of COVID-19 in the elderly explain these findings of significant and important effect sizes among older adults [38].

The potential underlying mechanisms that might explain how SARS-CoV-2 increases the risk of T1D are still being elucidated. As beta cells die or are destroyed, epitope spread contributes to a positive-feedback loop of increased activation of CD-8 T cells and production of an increasingly large population of autoantibodies to islet cells, insulin, glutamic acid decarboxylase, and protein tyrosine phosphatase [39]. As the population of functioning beta cells is depleted from these autoimmune insults, hyperglycemia and clinical T1D develops. As SARS CoV-2 gains entry to human cells via the ACE2 receptor, and this receptor has been recently shown to be expressed in the endocrine cells of the pancreatic islets [40], it is plausible that SARS CoV-2 may directly infect the human pancreas, although data on this are still emerging [41]. One recent study found that the pancreatic endocrine cells express many of the cell entry factors exploited by SARS-CoV-2 to infect human cells (including ACE2, TMPRSS2, NRP1, and TRFC), and that the virus is able to directly infect pancreatic beta cells in vitro [42]. Infected cells go on to produce less insulin, and ultimately the virus precipitates beta cell apoptosis (S1 Fig in S1 File) [42]. In this and a subsequent study, the authors additionally demonstrated SARS-CoV-2 RNA in the pancreatic beta cells of human autopsies of COVID-19 patients [43].

A similar dearth of studies has explored the impact of COVID-19 on T1D complications such as DKA. Early in the pandemic, a study out of Wuhan, China found preliminary evidence of increased risk of ketosis and DKA among those with pre-existing DM, although only one patient in this study was known to have T1D specifically [17]. Similar observations have been made in studies and case reports from India [22], Singapore [15], and the UK [44]. In a US study, it was also observed that severe DKA was dramatically more common among non-Hispanic Black patients relative to non-Hispanic White patients (OR 3.7, 95% CI: 1.4, 10.6), which may suggest that COVID-19 not only increases the incidence of DKA among the T1D population in the US, but also exacerbates inequalities in health for Black patients [45]. However, our study did not find statistically significant differences in odds of DKA among non-Hispanic Black patients when compared to non-Hispanic White patients.

Though the odds of DKA were not significantly increased among non-Hispanic Black patients, we did observe a statistically significant increase in odds of developing DKA following COVID-19 diagnosis in the overall population of patients with pre-existing T1D. DKA is a potentially fatal complication of DM, most commonly occurring in T1D [17] that is caused by insulin deficiency that results in excess ketone production. Though additional research on underlying mechanisms precipitating increased odds of DKA following COVID-19 diagnosis is needed, previous researchers have hypothesized that this effect could be driven by COVID-19-related insults to pancreatic beta cells [15] resulting in increased beta cell dysfunction [6]. SARS-CoV-2 utilizes the ACE2 receptor to enter host cells, and ACE2 has recently been shown to be expressed in pancreatic beta cells in addition to the lungs [40, 43, 44, 46]. The role of Interleukin-6 (IL-6) has also recently been investigated as a mechanistic explanation driving increased odds of DKA, as IL-6 levels are elevated in both DKA and COVID-19 [21], although this finding may only have prognostic relevance.

Our study has a number of important limitations. First, our cohort was restricted to patients within Cerner Health Systems, which may impact the generalizability of our findings. Notably, the majority of patients in our cohort are non-Hispanic White individuals, which indicates that our cohort may under-represent underserved minority populations. Similarly, most COVID-19 testing in the Cerner Health System was performed in an inpatient-emergency setting where higher risk, more severely symptomatic patients presented. Thus, our cohort was selective for those with higher rates of hospitalization and COVID-19 complications. Additionally, our analysis on incident T1D and COVID-19 assumes all patients in the cohort without qualifying codes truly had no T1D or COVID-19. Misclassification bias may be present if patients with T1D or COVID-19 were not accurately identified in Cerner prior to our analysis. It is possible that there was outcome misclassification in terms of diabetes type, with some patients presenting with T2D or other forms of diabetes miscoded as T1D and vice versa. We selected a short (1+ day lag) post-COVID-19 diagnosis window for identification of incident T1D cases given the dramatic and precipitious disease onsets described in the prior case reports. However, it is possible that this this led to inclusion of some participants who were developing T1D prior to COVID-19 diagnosis, and were identified when they presented for virus-related medical treatment. The ECI was applied for measuring patient comorbidities which, like Charlson Comorbidity Index (CCI) [47] is validated for adult populations. Although studies of T1D generally involve a higher prevalence of pediatric patients, and other studies [48] have shown that pediatric-specific comorbidity indices perform most optimally among these groups, this study population largely comprised adults. Lastly, because of the pandemic, many patients avoided hospital visits [49, 50]. This phenomenon impacted the availability of patient data beginning in March 2020. In attempts to account for this effect of the pandemic and capture more relevant clinical information, we increased the length of our inclusion window for non-COVID patients. Additionally, changes in healthcare availability and overcrowding of emergency rooms may have limited care for T1D patients. Thus, societal factors that could not be measured, in addition to the biologic factors investigated in this study, may have also contributed to development of DKA.

These limitations are counterbalanced by a number of important strengths. A major strength of this study is that the size and scope of the *Cerner Real-World Data* make it possible to analyze associations between T1D and COVID-19 on a national scale. This study is the first to utilize a national US cohort to examine the relationship between COVID-19 diagnosis and T1D incidence among those with no history of T1D. Additionally, the data made possible the investigation of the impact of COVID-19 diagnosis on the risk of DKA in patients with pre-existing T1D, an assessment which has never before been performed on such a large scale.

In conclusion, our study contributes to the limited, but expanding, literature on T1D in the context of COVID-19. Using data from a national US cohort, we found that the risk of new-onset T1D is significantly increased following COVID-19 diagnosis. Our results also highlight additional racial/ethnic disparities in COVID-19 outcomes and prognosis, as the risk of new-onset T1D is disproportionately high among American Indian/Alaskan Native, Asian/Pacific Islander, and Black populations. These findings emphasize the need to better support and meet the needs of underprivileged and underserved minority populations, especially within the US healthcare system. In addition to findings on T1D incidence, we observed that among patients with prior T1D diagnosis, the risk of developing DKA is significantly increased following COVID-19 diagnosis. Due to the increasing incidence and potentially fatal nature of DKA [20], prevention and timely diagnosis are critical. Thus, awareness of the heightened risk of DKA for T1D patients with COVID-19 diagnosis is essential as it can promote close monitoring and early intervention, leading to improved prognosis.

## Supporting information

S1 File (DOCX)
