# Supplementary material for: The associations between COVID-19 diagnosis, type 1 diabetes, and the risk of diabetic ketoacidosis: A nationwide cohort from the US using the Cerner Real-World Data
Source: PLoS One. 2022 Apr 19;17(4):e0266809. doi: 10.1371/journal.pone.0266809 (PMC9017888; doi:10.1371/journal.pone.0266809)
Supplement: S1 File — (DOCX) [file pone.0266809.s001.docx]

**Supplemental Table 1:** List of ICD-10 codes used to define T1D and DKA

| Chronic Disease | ICD-10 Codes |
| --- | --- |
| T1D | E10, E10.1, E10.10, E10.11, E10.2, E10.21, E10.22, E10.29,  E10.3, E10.31, E10.311, E10.319, E10.32, E10.321, E10.329, E10.33, E10.331,  E10.339, E10.34, E10.341, E10.349, E10.35, E10.351, E10.359, E10.36, E10.39,  E10.4, E10.40, E10.41, E10.42, E10.43, E10.44, E10.49, E10.5, E10.51, E10.52,  E10.59, E10.6, E10.61, E10.610, E10.618, E10.62, E10.620, E10.621, E10.622,  E10.628, E10.63, E10.630, E10.638, E10.64, E10.641, E10.649, E10.65, E10.69,  E10.8, E10.9, O24.0, O24.01, O24.011, O24.012, O24.013, O24.019, O24.02,  O24.03 |
| DKA | E10.1, E10.10, E10.11 |

**Supplemental Table 2:** List of chronic diseases involved in Elixhauser comorbidity index, with corresponding ICD-10 codes

| Chronic Disease | ICD-10 Codes |
| --- | --- |
| Congestive heart failure | I09.9, I11.0, I13.0, I13.2, I25.5, I42.0, I42.5 - I42.9, I43.x, I50.x, P29.0 |
| Cardiac arrhythmias | I44.1 - I44.3, I45.6, I45.9, I47.x - I49.x, R00.0, R00.1, R00.8, T82.1, Z45.0, Z95.0 |
| Valvular disease | A52.0, I05.x - I08.x, I09.1, I09.8, I34.x - I39.x, Q23.0 - Q23.3, Z95.2 - Z95.4 |
| Pulmonary circulation disorders | I26.x, I27.x, I28.0, I28.8, I28.9 |
| Peripheral vascular disorders | I70.x, I71.x, I73.1, I73.8, I73.9, I77.1, I79.0, I79.2, K55.1, K55.8, K55.9, Z95.8, Z95.9 |
| Hypertension: uncomplicated | I10.x |
| Hypertension: complicated | I11.x - I13.x, I15.x |
| Paralysis | G04.1, G11.4, G80.1, G80.2, G81.x, G82.x, G83.0 - G83.4, G83.9 |
| Other neurological disorders | G10.x - G13.x, G20.x - G22.x, G25.4, G25.5, G31.2, G31.8, G31.9, G32.x, G35.x - G37.x, G40.x, G41.x, G93.1, G93.4, R47.0, R56.x |
| Chronic pulmonary disease | I27.8, I27.9, J40.x - J47.x, J60.x - J67.x, J68.4, J70.1, J70.3 |
| Diabetes, uncomplicated | E10.0, E10.1, E10.9, E11.0, E11.1, E11.9, E12.0, E12.1, E12.9, E13.0, E13.1, E13.9, E14.0, E14.1, E14.9 |
| Diabetes, complicated | E10.2 - E10.8, E11.2 - E11.8, E12.2 - E12.8, E13.2 - E13.8, E14.2 - E14.8 |
| Hypothyroidism | E00.x - E03.x, E89.0 |
| Renal failure | I12.0, I13.1, N18.x, N19.x, N25.0, Z49.0 - Z49.2, Z94.0, Z99.2 |
| Liver disease | B18.x, I85.x, I86.4, I98.2, K70.x, K71.1, K71.3 - K71.5, K71.7, K72.x - K74.x, K76.0, K76.2 - K76.9, Z94.4 |
| Peptic ulcer disease, excluding bleeding | K25.7, K25.9, K26.7, K26.9, K27.7, K27.9, K28.7, K28.9 |
| AIDS/HIV | B20.x - B22.x, B24.x |
| Lymphoma | C81.x - C85.x, C88.x, C96.x, C90.0, C90.2 |
| Metastatic cancer | C77.x-C80.x |
| Solid tumour without metastasis | C00.x - C26.x, C30.x - C34.x, C37.x - C41.x, C43.x, C45.x - C58.x, C60.x - C76.x, C97.x |
| Rheumatoid arthritis/collagen vascular diseases | L94.0, L94.1, L94.3, M05.x, M06.x, M08.x, M12.0, M12.3, M30.x, M31.0 - M31.3, M32.x - M35.x, M45.x, M46.1, M46.8, M46.9 |
| Coagulopathy | D65 - D68.x, D69.1, D69.3 - D69.6 |
| Obesity | E66.x |
| Weight loss | E40.x - E46.x, R63.4, R64 |
| Fluid and electrolyte disorders | E22.2, E86.x, E87.x |
| Blood loss anaemia | D50.0 |
| Deficiency anaemia | D50.8, D50.9, D51.x - D53.x |
| Alcohol abuse | F10, E52, G62.1, I42.6, K29.2, K70.0, K70.3, K70.9, T51.x, Z50.2, Z71.4, Z72.1 |
| Drug abuse | F11.x - F16.x, F18.x, F19.x, Z71.5, Z72.2 |
| Psychoses | F20.x, F22.x - F25.x, F28.x, F29.x, F30.2, F31.2, F31.5 |
| Depression | F20.4, F31.3 - F31.5, F32.x, F33.x, F34.1, F41.2, F43.2 |

**Supplemental Table 3:** List of codes used to define continuous glucose monitoring (CGM) and insulin pump usage

| Variable | Code Type | Codes |
| --- | --- | --- |
| CGM |  |  |
|  | HCPCS | A9277, S1036, A9276, S1035, A9278, S1037 |
|  | CPT | 95249, 95250, 95251, 99212, 99213, 99214, 99215, 99417, G2212 |
| Insulin pump |  |  |
|  | ICD-10 | Z79.4 |
|  | HCPCS | E0784, S1034, A4230, A4231, A4232, A4224, A4225, J1815, J1817 |


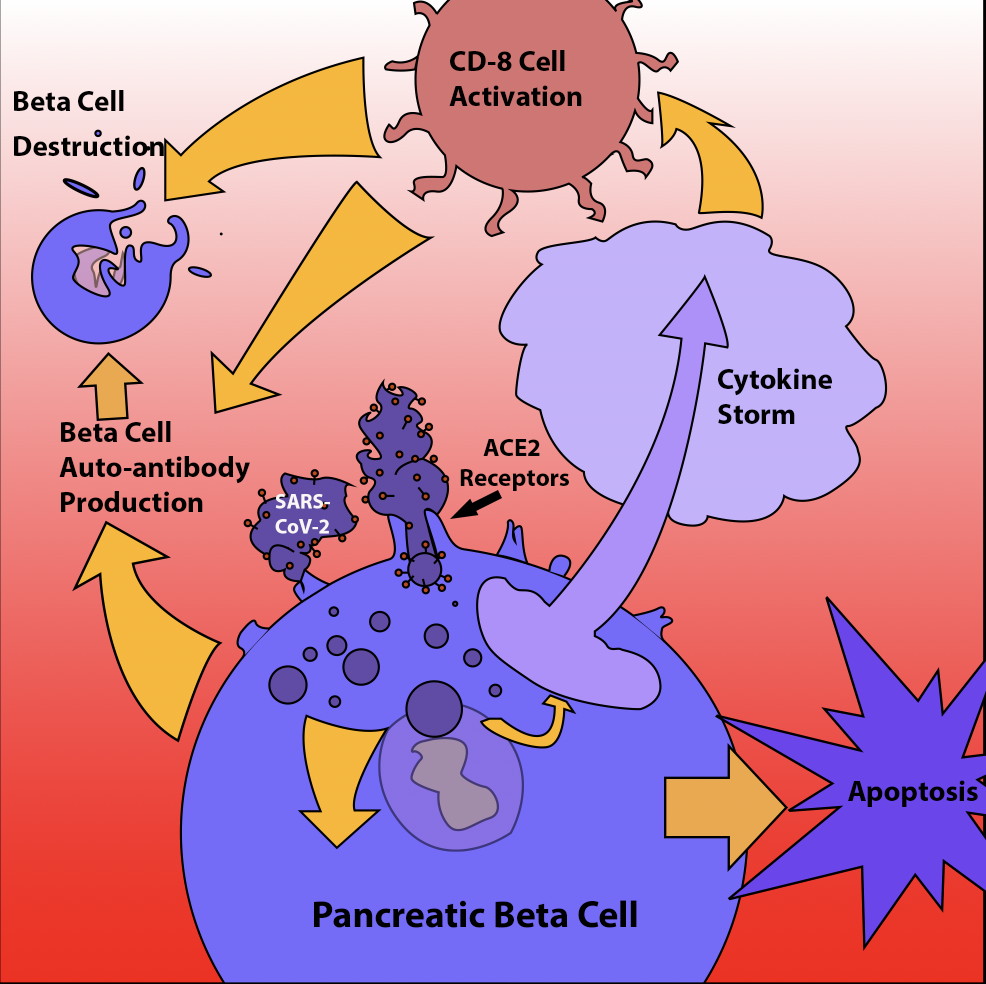


**Supplemental Figure 1: Potential biological mechanisms that might explain how SARS-CoV-2 increases the risk of T1D.** SARS-CoV-2 gains entry to pancreatic beta cells via the ACE2 receptor. Infected cells go on to produce less insulin, and ultimately the virus precipitates apoptosis [31]. As beta cells die, epitope spread contributes to increased activation of CD-8 T cells and production of beta cell autoantibodies [14]. As the population of functioning beta cells is depleted from these autoimmune insults, hyperglycemia and clinical T1D develops.

**Supplemental Table 4:** Adjusted associations of COVID-19 diagnosis with incidence of DKA among patients with previous T1D diagnosis (complete cases and removal of HbA1c from model)

|  | Complete Cases | | Without HbA1c adjustment | |
| --- | --- | --- | --- | --- |
|  | **aOR^*^ (95% CI)** | **p-value** | **aOR^*^ (95% CI)** | **p-value** |
| COVID-19 |  |  |  |  |
| No | REF=1 | - | REF=1 | - |
| Yes | 2.61 (2.18, 3.13) | **<0.001** | 2.62 (2.37, 2.89) | **<0.001** |
|  |  |  |  |  |
| Age | 0.97 (0.96, 0.98) | **<0.001** | 0.96 (0.95, 0.97) | **<0.001** |
|  |  |  |  |  |
| Gender |  |  |  |  |
| Female | REF=1 | - | REF=1 | - |
| Male | 0.90 (0.77, 1.04) | 0.15 | 0.98 (0.90, 1.07) | 0.62 |
|  |  |  |  |  |
| Race and Ethnicity |  |  |  |  |
| NH-White | REF=1 | - | REF=1 | - |
| NH-AI/AN | 0.79 (0.41, 1.54) | 0.49 | 0.85 (0.59, 1.23) | 0.40 |
| NH-API | 0.68 (0.32, 1.47) | 0.33 | 0.72 (0.49, 1.06) | 0.10 |
| NH-Black | 0.97 (0.77, 1.23) | 0.82 | 1.07 (0.94, 1.22) | 0.33 |
| Hispanic/Latino | 0.87 (0.70, 1.07) | 0.19 | 0.81 (0.72, 0.92) | **0.001** |
| NH-Other | 0.68 (0.49, 0.95) | **0.02** | 0.65 (0.53, 0.78) | **<0.001** |
|  |  |  |  |  |
| Marital Status |  |  |  |  |
| Married/Partner | REF=1 | - | REF=1 | - |
| Not Married | 1.23 (0.97, 1.55) | 0.09 | 1.31 (1.15, 1.49) | **<0.001** |
|  |  |  |  |  |
| Region |  |  |  |  |
| Northeast | REF=1 | - | REF=1 | - |
| Southeast | 0.83 (0.63, 1.09) | 0.18 | 0.66 (0.57, 0.76) | **<0.001** |
| Midwest | 0.89 (0.69, 1.15) | 0.38 | 0.70 (0.61, 0.80) | **<0.001** |
| West | 1.39 (1.09, 1.77) | **0.01** | 1.32 (1.17, 1.48) | **<0.001** |
|  |  |  |  |  |
| ECI | 1.02 (1.01, 1.03) | **<0.001** | 1.02 (1.01, 1.03) | **<0.001** |
|  |  |  |  |  |
| Insulin pump |  |  |  |  |
| No | REF=1 | - | REF=1 | - |
| Yes | 0.36 (0.31, 0.43) | **<0.001** | 0.44 (0.40, 0.48) | **<0.001** |
|  |  |  |  |  |
| Continuous glucose monitoring |  |  |  |  |
| No | REF=1 | - | REF=1 | - |
| Yes | 0.85 (0.65, 1.12) | 0.24 | 0.75 (0.62, 0.91) | **0.002** |
|  |  |  |  |  |
| Duration of diabetes (Years) | 0.99 (0.97, 1.01) | 0.27 | 1.00 (0.99, 1.01) | 0.59 |
|  |  |  |  |  |
| Baseline HbA1c | 1.23 (1.19, 1.27) | **<0.001** | - | **-** |

^*^ adjusted odds ratio
